# Pavilion Lake Microbialites: Morphological, Molecular and Biochemical Evidence for a Cold-Water Transition to Colonial Aggregates

**DOI:** 10.3390/life3010021

**Published:** 2012-12-27

**Authors:** Dirk Schulze-Makuch, Darlene Lim, Bernard Laval, Carol Turse, Marina Resendes de Sousa António, Olivia Chan, Stephen B. Pointing, Allyson Brady, Donnie Reid, Louis N. Irwin

**Affiliations:** 1 School of the Environment, Washington State University, Pullman, WA 99164, USA; E-Mails: carol@turse.org (C.T.); Antonio.Marina@mayo.edu (M.R.D.S.A.); 2 NASA Ames Research Center, Mail-Stop 245-3, Moffett Field, CA 94035, USA; E-Mail: darlene.lim@nasa.gov; 3 Department of Civil Engineering, University of British Columbia, Vancouver, BC, V6T 1Z4, Canada; E-Mail: blaval@mail.ubc.ca; 4 Mayo Clinic, Medical Sciences Building, 321 Third Avenue SW, Rochester, MN 55905, USA; 5 School of Biological Sciences, The University of Hongkong, Hongkong; E-Mails: chanolivia@gmail.com (O.C.); pointing.steve@gmail.com (S.B.P.); 6 Department of Biological Sciences, University of Calgary, 2500 University Drive NW, Calgary, Alberta, T2N 1N4, Canada; E-Mail: albrady@ucalgary.ca; 7 Nuytco Research Ltd, 216 East Esplanade, North Vancouver, BC, V7L 1A3, Canada; E-Mail: donnie@nuytco.com; 8 Department of Biological Sciences, University of Texas at El Paso, TX 79968, USA; E-Mail: lirwin@utep.edu

**Keywords:** microbialite, community, stromatolite, quorum sensing, biomass, biodiversity, ecology

## Abstract

The presence of microbialite structures in a freshwater, dimictic mid-latitude lake and their establishment after the last ice age about 10,000 years ago is puzzling. Freshwater calcite microbialites at Pavilion Lake, British Columbia, Canada, consist of a complex community of microorganisms that collectively form large, ordered structured aggregates. This distinctive assemblage of freshwater calcite microbialites was studied through standard microbial methods, morphological observations, phospholipid fatty acid (PLFA) analysis, DNA sequencing and the identification of quorum sensing molecules. Our results suggest that the microbialites may represent a transitional form from the exclusively prokaryotic colonial precursors of stromatolites to the multicellular organismic aggregates that give rise to coral reefs.

## 1. Introduction

Pavilion Lake (50°51'57"N, 121°44'20"W), located in Marble Canyon in the interior of British Columbia, Canada, is 5.7 km long and has an average width of 0.8 km. A detailed description of the geological and hydrological setting of Pavilion Lake is given in Lim *et al.* [1], but a brief synthesis is provided herein. Pavilion Lake is a slightly alkaline (mean pH = 8.3), freshwater lake with a maximum-recorded depth of 65m. Water temperatures at Pavilion Lake range between 0 and 20 °C, but are usually below 10 °C at the depths where the microbialites form. The lake was added to the Marble Canyon B.C. Provincial Park system on April 18, 2001, as a means of conserving and managing this biologically and historically important site. The lake hosts unusual, large (meter scale) freshwater carbonate structures referred to as microbialites due to the hypothesized biological role in their formation [2]. Evidence of biological influences on carbonate precipitation within micro-stromatolitic surface nodules associated with the microbialites has been identified using ^13^C isotopic biosignatures [3]. Understanding the physiochemical and biological controls on microbialite morphogenesis in Pavilion Lake could provide significant insights into the interpretation of late Proterozoic to early Paleozoic reef structures, and as such, a multi-disciplinary project is currently underway (see Pavilion Lake Research Project (PLRP) website (http://PavilionLake.com) to meet these scientific objectives. 

## 2. Observations

### 2.1. Morphology

**Figure 1:**
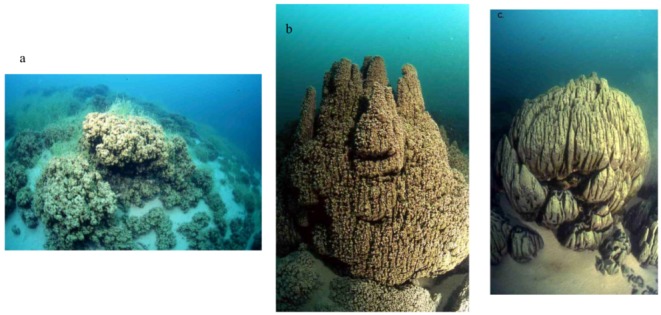
Examples of varying microbialite morphologies in Pavilion Lake. (1a) Shallow to Upper Intermediate (10–15 m). (1b) Intermediate to Lower Intermediate (about 20 m). (1c) Deep Water (25–35 m).

Laval *et al.* [2] described microbialite reefs oriented perpendicularly to the shoreline from depths of 5–30 m in selected regions of Pavilion Lake; however, recent Multi-Angle Swath Bathymetry (MASB) data, comprehensive pilot and video observations from manned DeepWorker submersibles and remotely operated vehicle borne camera observations have demonstrated that microbialite coverage in the lake is more extensive than originally hypothesized [4]. They are found throughout the lake basin and on top of mounds at depths of 45–60 m [5]. The microbialites were originally categorized into three morphological groupings by depth: those found at shallow to intermediate depth of 10–15 m (Figure 1a), cone-shaped seepage structure with hollow internal conduits that open at the top of the cones at intermediate depths of about 20 m (Figure 1b), and artichoke-type mounds, centimeters to meters in diameter at depths ranging from 25 to 35 m (Figure 1c). The mounds found at the shallow to upper intermediate depths (<15 m) range from several centimeters to a few decimeters in height and are noted to be covered by photosynthetic microbial communities and their calcified remains. At the intermediate depth of approximately 20 m, carbonate domes approximately decimeters to meters in diameter and up to 3m in height were documented and are likely the largest structures in the lake. At the lower intermediate to deeper depths of 25–35 m, the morphology of the carbonates can be described as a combination of “cone-shaped and leaf-like” [2]. These structures are denser than those at the shallower depths and are typically 20–30 cm in height and decimeters to meters in diameter. These structures are sometimes capped by ‘chimney like’ formations that range in height from 5–30 cm (e.g., Figure 1b). 

**Figure 2:**
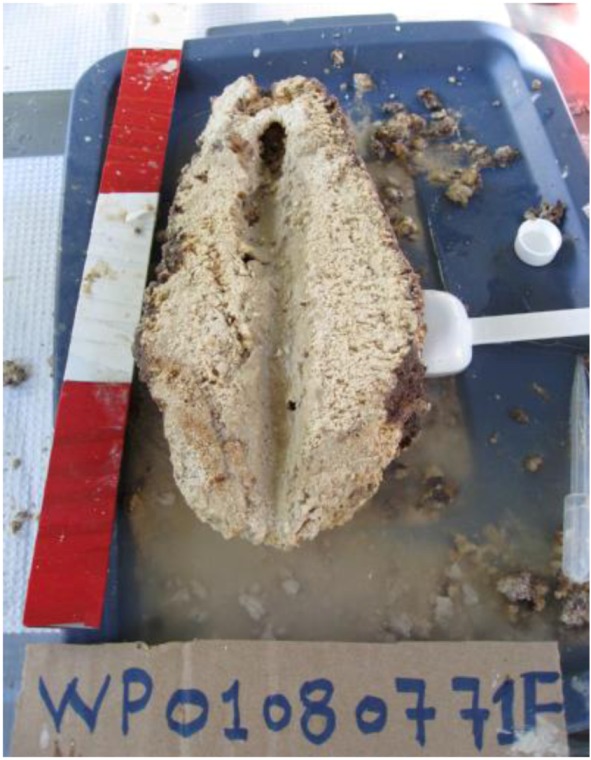
Internal structure of ‘chimneys’ structure from sample obtained August 2007.

A single internal conduit is seen in all sampled cases, running the length of these ‘chimneys’ (Figure 2). These conduits are filled with fine-grained carbonate sediment. It is unclear at this time what function or environmental correlation these structures may have with groundwater inputs into Pavilion Lake. Finally, the deepest characterized carbonates were reported at depths of 30–35 m, although we now know this lower limit to be much deeper at 45–55 m [4]. Overall, the mechanical strength of the structures increases with depth, which may reflect a change in the relative role of biotic and non-biotic precipitation with lowering light levels. 

To establish approximate ages and rates of growth more precisely, six ^230^Th/^234^U analyses were made by Laval *et al.* [2] of the calcite in the lower to upper-middle parts of two whole mounds (collected from depths of 27 and 32 m) using thermal ionization mass spectrometry (TIMS), plus one bulk analysis by alpha spectrometry of the center of a third mound (collected from 32 m depth). Raw ages that ranged from 12,300 ± 1,400 to 3,650 ± 860 years were derived and interpreted to represent the maximum possible age of the carbonates. As such, these structures appear to have begun formation nearly 11,000 years ago, after the glacial retreat of the Cordilleran Ice Sheet. Based upon these age estimates, they extended at rates between 2.5 and 3.0 cm per thousand years. Analysis of microbialite associated with a detrital branch within Pavilion Lake using ^14^C has identified growth estimates of 0.05 mm per year within the past ~ 1,000 years [6]. This rate is faster than the previous estimates, indicating that some variation in microbialite growth rate exists within this system.

### 2.2. Biochemistry

**Figure 3:**
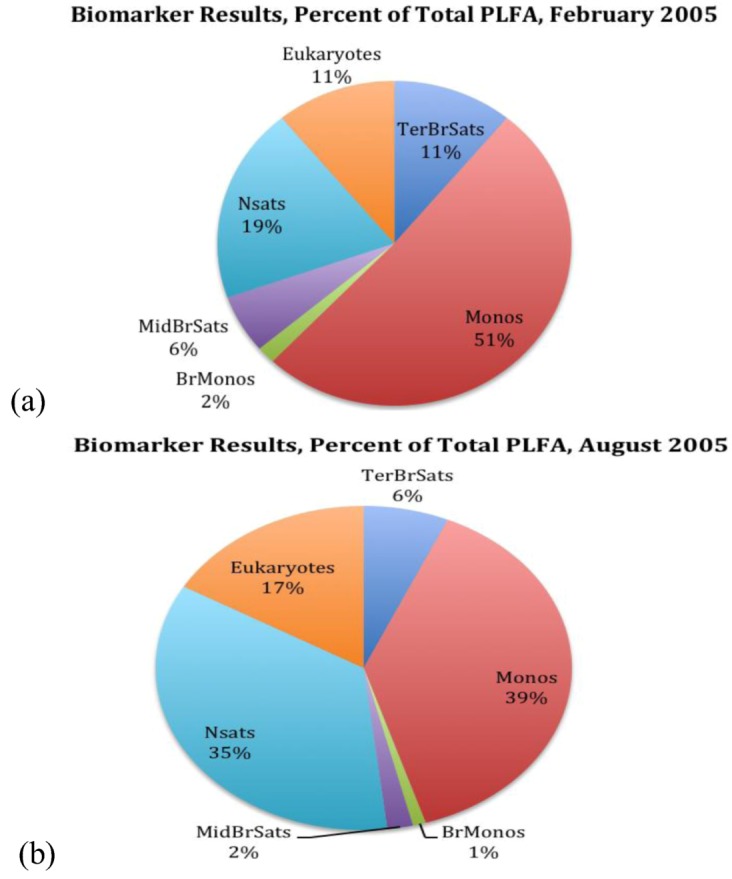
Biomarker results (%) from scraped off microbialite sample FPav-Microb and APav-Microb in percent of total phospholipid fatty acid (PLFA), as sampled in (a) February 2005 and (b) August 2005. The abbreviations are as follows: Terminally branched saturates (TerBrSats), Monoenoics (Monos), Branched monoenoics (BrMonos), Mid-chain branched saturates (MidBrSats) and Normal saturates (NSats).

**Figure 4:**
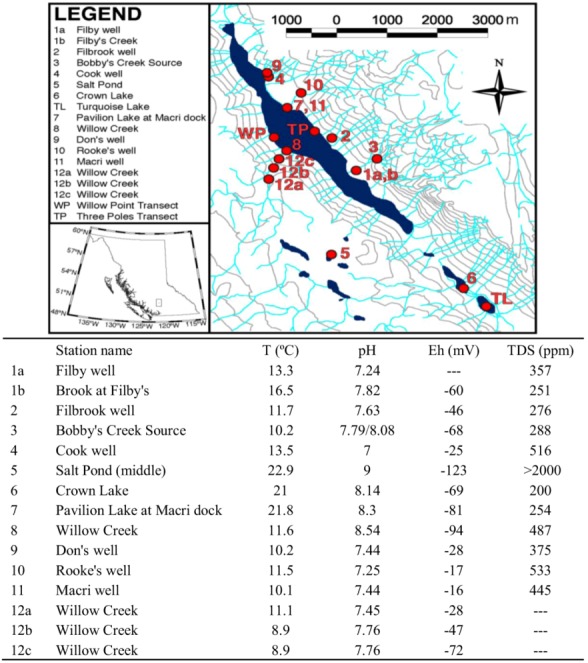
Pavilion Lake, overview map. Field parameters as indicated were measured in August 2005. The streams marked in light blue are ephemeral.

Assays were carried out on a total of 14 water samples and two microbialite samples at or near Pavilion Lake by phospholipid fatty acid (PLFA) analysis. Samples were taken in February and August 2005. In February, one sample was scraped off from a deep microbialite structure (artichoke type, see Figure 1c; FPav-Microb) at Willow Point, one of two transect locations within the lake that are a focus of the PLRP study. Further water samples were obtained from next to that structure (FPav-Deep) and next to the medium (chimney-type, Figure 1b) and shallow microbialites (Figure 1a; FPav-Medium and FPav-Shallow, respectively, each sample taken about 1 m from the structures). Another sample was taken from the open water at Pavilion Lake (FPav-OpenW). In August of the same year, another sample was scraped off from a deep microbialite at Willow Point (APav-Microb) to evaluate seasonal effects on the community structure (Figure 3a,b; Table 1). An additional total of 10 samples were collected at and near Pavilion Lake, as shown in Figure 4.

The PLFA analysis was used to measure viable microbial biomass, community composition and nutritional status [7]. Phospholipid fatty acids are a component of cellular membranes that maintain cell fluidity, enabling the transport of nutrients into the cell and the elimination of by-products. The total phospholipid ester-linked fatty acids provide a quantitative measure of viable or potentially viable biomass, because cellular enzymes hydrolyze and release the phosphate group within minutes to hours of cell death. PLFAs are not only viable biomass indicators, but also are suitable taxonomic markers, because different groups of microbes synthesize a variety of PLFAs through various biochemical pathways [8]. These correlations can be very strong, so that fatty acid biomarkers have been identified for particular organisms [7,9,10,11,12]. The lipids were analyzed using the modified Bligh and Dyer method, as described by Smith *et al.* [13], Schulze-Makuch and Kennedy [14] and Schulze-Makuch *et al.* [15]. 

In summary, lipids were recovered, dissolved in chloroform and fractionated on disposable silicic acid columns into neutral-, glyco- and polar-lipid fractions. The polar lipid fraction was trans-esterified with mild alkali to obtain the PLFA as methyl esters in hexane. PLFAs were then analyzed by gas chromatography with peak conformation performed by electron impact mass spectrometry (GC/MS). A filter blank was used as negative control to ensure no laboratory contamination [8]. The methodology used differentiates between six structure groups: terminally branched saturated, monoenoics, branched monoenoic, mid-chain branched saturated, normal saturated and eukaryotic PLFAs. Terminally branched saturated PLFAs are characteristic of gram-positive bacteria, but also occur in the cell membranes of many sulfate reducing bacteria. Typically, gram-positive bacteria grow slower than gram-negative bacteria and are capable of degrading more complex compounds. Monoenoic PLFA is indicative of gram-negative bacteria, which are fast growing, use many carbon sources and adapt quickly to a variety of environments. Branched monoenoics often occur in the cell membranes of obligate anaerobes, such as iron or sulfate reducing bacteria. Mid-chain branched saturated PLFAs are common in *Actinomycete spp*., sulfate-reducing bacteria and certain gram-positive bacteria. Normal saturated PLFAs occur in both the prokaryotic and eukaryotic organisms. High concentrations of normal saturated PLFAs commonly occur in less diverse populations. Polyunsaturated PLFAs (PUFA) are generally considered typical for eukaryotes, which include protozoa, fungi, algae, plants and animals. However, some 18:2 and 18:3 biomarkers are produced by cyanobacteria [16,17,18] and have been attributed to cyanobacteria in previous studies [19,20]. Polyunsaturated PLFA have also been identified in cyanobacteria dominated nodule communities associated with the Pavilion Lake microbialite surfaces [3]. In addition to these structure groups, several biomarkers were identified, which are typical for specific prokaryotic and eukaryotic microbes (Table 1). For example, the biomarker 20:5w3 has a total of 20 carbons in the fatty acid, five double bonds form the aliphatic (w) end of the molecule and the third carbon is positioned from the aliphatic end before the double bond. Biomarker 20:5w3 is indicative for diatoms.

**Table 1:** Major selected biomarker for each category of microorganism, based on Figure 3 (total includes minor biomarkers not shown; see also Figure 3 legend for abbreviations used).

Biomarkers	ECL^1^	February’05	August’05
Terminally Branched Saturates (TerBrSats)			
i15:0	14.64	2.6	2.1
a17:0	16.73	2.5	1
Total		11	6.6
Monoenoics (Monos)			
16:1w7c	15.77	17.9	10.4
18:1w7c	17.79	13	15.9
Total		51.1	38.6
Branched Monoenoics (BrMonos)			
i17:1w7c	16.38	1.1	0.4
br19:1a	18.05	0.6	0.6
Total		1.7	1
Mid-Chain Branched Saturates (MidBrSats)			
10me16:0	16.45	3.9	1.4
12me16:0	16.48	1.6	0.5
Total:		5.8	2
Normal Saturates (NSats)			
14:00	14	0.7	6.2
16:00	16	15.4	26.8
Total:		19.4	35.2
Eukaryotes			
18:2w6	17.64	3.6	7.3
18:3w3	17.68	2.6	6.3
20:5w3	19.32	3.3	1.8
Total		11	16.6

^1^ ECL = Equivalent Chain Length. Note: The PLFA nomenclature is based upon the number of carbons in the molecule (first number) and the number of double bonds following the colon. The prefixes “a” and “i” refer to antiiso- and iso-branched fatty acids, “me” refers to a midchain methyl group, “cy” to a cyclopropyl group, “c” stands for cis-configuration, “t” for trans-configuration. For example, the biomarker 20:4w6 has a total of 20 carbons in the fatty acid and 4 double bonds from the aliphatic [w] end of the molecule, and the 6^th^ carbon atom is positioned from the aliphatic end before the double bond. This biomarker is characteristic for protozoa [8].

PLFA biomarkers were also used to evaluate the metabolic status of the gram-negative microbial population in Pavilion Lake. In monoenoics, the biomarkers 16:1w7c and 18:1w7c (c for cis configuration) are converted to cyclopropyl fatty acids (cy17:0 and cy19:0) as microbial cells move from a log phase to a stationary phase of growth. This change is expressed as ratios. The ratios vary, depending on the organism and the environment, but generally fall within the range from 0.05 (log phase) to 2.5 (stationary phase). The ratio is inversely proportional to the turnover rate and a lower ratio implies a higher turnover rate [21,22]. The analyzed samples from Pavilion Lake indicate stationary growth and a low turnover rate. Biomarkers can also be used to detect environmental stress in microbes. Gram-negative bacteria generate trans-fatty acids to minimize the permeability of their cell membranes as protection against changes in the environment, such as starvation or toxicity. The ratios 16:1w7t/16:1w7c (t for trans configuration) and 18:1w7t/18:1w7c have been shown to indicate the effects of starvation on bacterial isolates. The range is generally between 0.05 (healthy) to 0.3 (starved) or 0.1 (healthy) to 0.6 (starved) when the two ratios are added [23]. The analyzed samples from Pavilion Lake did not indicate any evidence of starvation.

**Figure 5:**
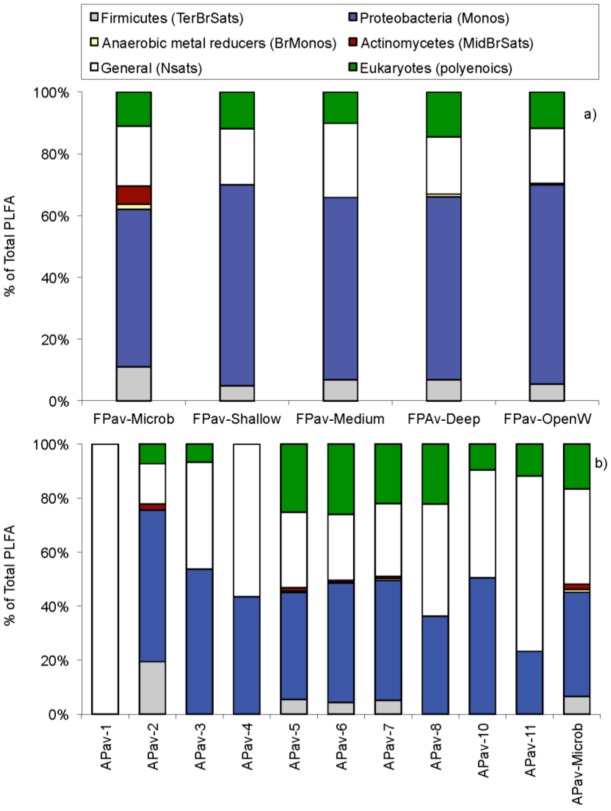
Deduced community structure based on PFLA analysis at or near Pavilion Lake. (5a) sampling round in Pavilion Lake with sample FPav-Microb scraped off from a microbialite, shallow, medium and deep samples corresponding to the depth levels at which those respective microbialites occur (sampled about 1 m away from the structures) and open water resembling a near-surface water sample at Pavilion Lake. (5b) Sampling round with APav-Microb scraped off from a microbialite with other designations as in Figure 4.

Ground-water samples generally exhibited less diverse microbial communities dominated by normal saturated and monoenoic saturated PLFA (Figure 5b). More diversity was observed in lake samples (APav-5,6 & 7), but branched monoenoic PLFA, which can be typical for sulfate and iron reducing bacteria, was only observed in samples from Pavilion Lake (APav-7 & APav-Microb). The concentration of branched monoenoic PLFA was present at higher concentrations (both relative and in total amount) in sample APav-Microb, which was scraped off from a microbialite. In fact, the largest diversity was observed in this sample, indicating a highly diverse microbial population associated with the microbialites. A diverse suite of micro-organisms, including biomarkers for sulfate reducers and anaerobic metal reducers, was also found in the microbialite sample FPav-Microb (Figure 5a). These results suggest that the diversity of organisms indicated in sample APav-Microb derive from the microbialites themselves and not the lake. The result of a diverse microbial population associated with the microbialites was further confirmed by an analysis for biomarkers at two different time periods, which revealed molecular biomarkers for proteobacteria, sulphur reducing bacteria and firmicutes (possibly photosynthetic heliobacteria), among others (Table 1). 

### 2.3. Biomass

**Figure 6:**
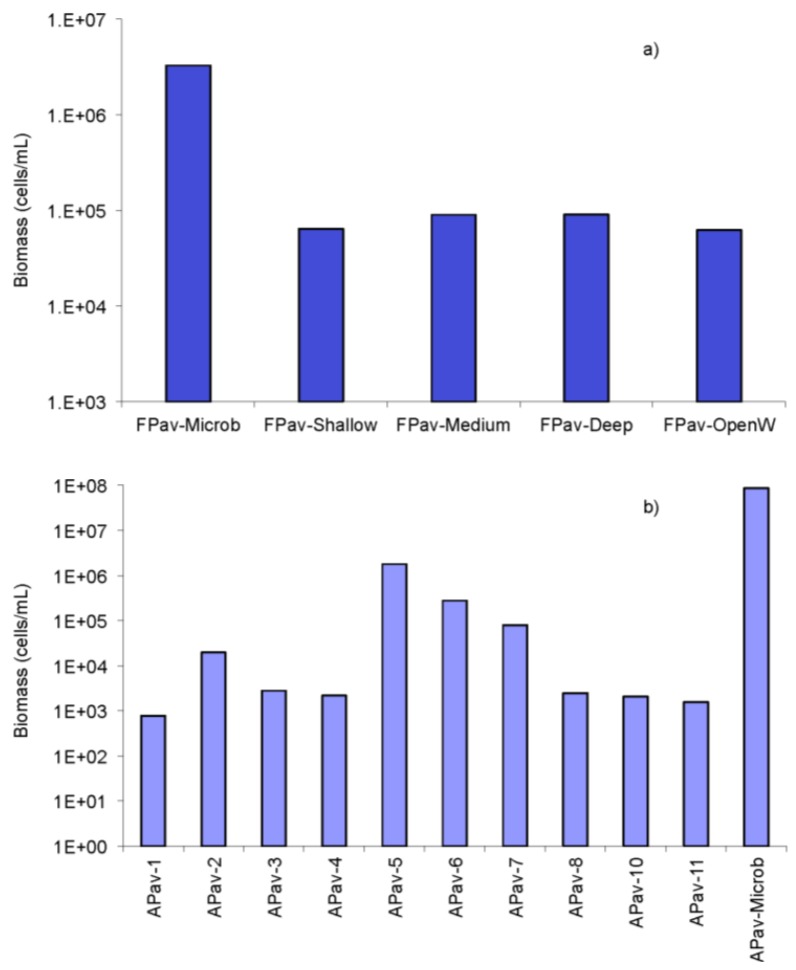
Biomass of various samples at and near Pavilion Lake. (6a) sampling round in Pavilion Lake with sample FPav-Microb scraped off from a microbialite, shallow, medium and deep samples corresponding to the depth levels at which those respective microbialites occur (sampled about 1 m away from the structures) and open water resembling a near-surface water sample at Pavilion Lake. (6b) Sampling round with APav-Microb scraped off from a microbialite with other designations as in Figure 4.

PLFA analysis is a reliable and accurate method to determine viable microbial biomass. It does not count dead or fossilized cells, since phospholipids break down rapidly upon cell death [24,25]. The sum of the PLFA is assumed to be proportional to the number of cells using a proportionality factor of 20,000 cells per picomole. The proportionality factor is taken from cells grown in the laboratory and exhibits some variation depending on the type of organism and environmental conditions [26]. Biomass samples reveal a somewhat larger biomass in the lake samples compared to ground-water samples. The largest biomass is observed from samples FPav-Microb and APav-Microb, which were scraped off from the microbialites. Their biomass is at least one order of magnitude higher than the measured biomasses in any other collected water sample (Figure 6). 

### 2.4. Community Structure

A molecular approach was used to assess spatio-temporal variation in microbial diversity of microbialites. This overcomes to some extent the bias associated with morphological and cultivation-based strategies for estimation of diversity. We used real-time quantitative polymerase chain reaction (Q-PCR) to estimate absolute and relative abundance of archaea, bacteria and eukarya using domain-specific rRNA gene primers. In order to establish the taxonomic composition of microbialites, we used terminal restriction fragment length polymorphism analysis (T-RFLP) to characterize occurrence of rDNA phylotypes, which were assigned a phylogenetic identity based upon sequence analysis of clone libraries. 

**Figure 7:**
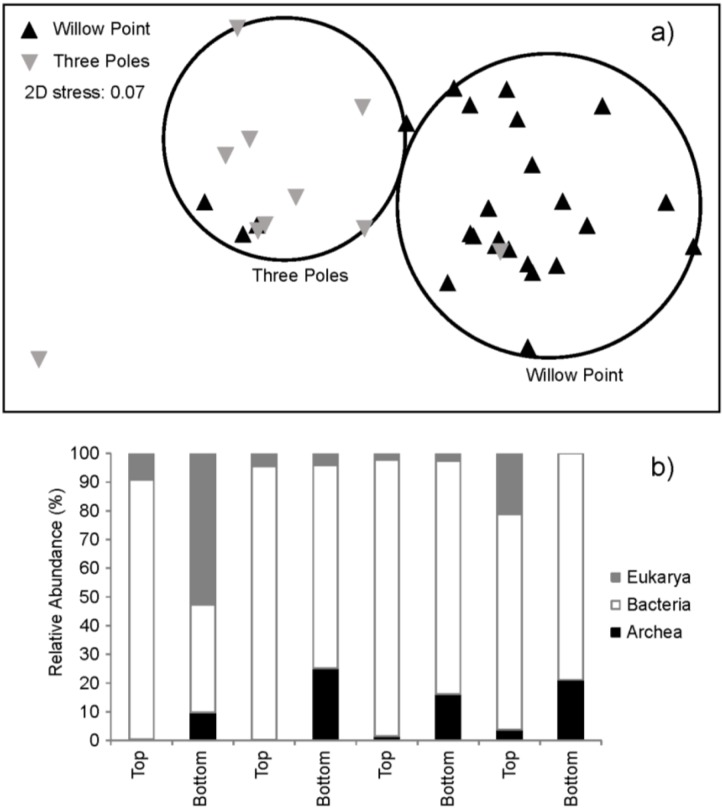
Variation in microbial community structure between microbialites recovered from Three Poles (TP) and Willow Point (WP) in Pavilion Lake. (7a) The differences in community structure as determined by sequence-based identification of rDNA phylotypes are illustrated by the ordinations of Bray Curtis similarity for communities from Three Poles and Willow Point, (7b) Bar chart illustrating differences in relative abundance of archaeal, bacterial and eukaryal rDNA signatures from active growing microbialites (top) and older structures (bottom) at various depths and locations.

Samples were analyzed from the top of the microbialite structures, likely indicating more recent growth, and from the bottom, where microbialites were in contact with the sediment, and likely indicate long established structures. The Q-PCR data revealed that rDNA signatures were an order of magnitude higher in young microbialites exposed to the water column compared to older structures on the lake bed. This suggests that biomass may be concentrated in the upper part of microbialites, whilst older microbialite structures may be relatively inert biologically. The Q-PCR data indicated that bacteria dominate the community in both locations, typically comprising over 90% of recoverable rDNA signatures (Figure 7b). Eukarya and archaea accounted for a comparatively low abundance of rDNA signatures in young microbialites. An interesting observation was that the older microbialite structures at the lake bed supported markedly higher abundance of archaeal signatures compared to those in the water column (Figure 7b). 

In order to start to understand spatial variability in diversity, we compared two locations, Three Poles and Willow Point, where long-term study transects have been established by the PLRP team (Figure 4). These two sites are on opposite sides of the central Pavilion Lake basin. Approximately 0.8 km separates the two transect locations. Differences in rDNA-defined diversity were assessed using T-RFLP. A non-metric multi dimensional scaling of Bray Curtis Similarity for bacterial diversity in each sample revealed two clusters broadly indicative of location (Figure 7a). This suggests that the microbialites at opposite sides of the basin may have developed distinct communities. Abiotic factors, such as UV radiation and groundwater seepage, may contribute to such variation, but this remains untested. Sequence analysis from clone libraries constructed for each location revealed that major differences could be accounted for by relative abundance of betaproteobacteria. The cyanobacterial genera *Pseudoanabaena*, *Oscillatoria* and *Calothrix*, which were previously reported from morphological studies by Laval *et al.* [2], were also present as rDNA signatures in this molecular survey. Furthermore, *Leptolyngbya*, a common cyanobacterial genus identified in marine stromatolites in Highborne Cay, Bahamas and Shark Bay, Western Australia and also recorded as an abundant morphotype in Pavilion Lake microbialites [27,28], was present in both libraries. 

### 2.5. Analysis of Quorum Sensing Molecules

Quorum sensing molecules are chemical signals that bacteria use to communicate and coordinate their activities via either diffusion to the extracellular environment or release into the intracellular environment. A wide range of diffusible signals that act extracellularly or are attached to the outside membrane of the cell is currently recognized (Table 2).

The term ‘quorum sensing’ is used to describe the system by which bacterial cells detect inducing levels of a chemical signal that is used to keep the bacterial population at a minimal level or quorum [29]. The small chemical signaling molecules that induce the quorum are called autoinducers (AI) and are important in such processes as antibiotic production, production of virulence factors, conjugation and transformation [30,31]. Quorum sensing can take place between bacteria of the same species, as well as between bacteria of different species. Additionally, some bacteria unable to synthesize their own signal are able to recognize the signals of other bacteria [32]. 

Wagner-Döbler *et al.* [33] screened over 100 bacterial isolates from various marine habitats for the presence of the bacterial cell signaling autoinducer molecule acyl homoserine lactone (AHL) with the result that about 40% of these isolates contained the AHL molecule. The range of bacterial isolates included Alpha-Proteobacteria, Gamma-Proteobacteria and gram-negative bacteria. Autoinducer-2 (AI-2), a furanosyl diester made from the reprocessing of S-adenosyl-homocysteine (SAH) to homocysteine, has also been studied as a possible interspecies communication signal [34]. S-adenosyl-homocysteine (SAH) can be recycled by a one-step or two-step process. The two-step process involves enzymatic conversion by the Pfs and LuxS enzymes, and the one-step process uses SAH-hydrolase (SahH). The researchers studied 138 complete genomes for genes involved in the synthesis and detection of AI-2 and found them in all cases except in some symbionts and parasites. Many Gamma-, Beta- and Epsilon-Proteobacteria and firmicutes possessed the two-step Pfs-LuxS pathway, while archaea, eukarya, Alpha-Proteobacteria, actinobacteria and cyanobacteria contained the one-step SahH pathway. Also, most proteobacteria and firmicutes contained the two-component sensor kinase protein LuxQ and the terminal response regulator LuxO. These are all microorganisms that were abundantly associated with the microbialites at Pavilion Lake. And indeed, when using degenerative primers and touchdown PCR for the quorum sensing molecules, LuxS and SahH genes were found in the intermediate depth microbialite samples from Pavilion Lake. We identified the LuxS gene sequence in a sample that derived from a depth of 23 meters and the SahH gene sequence in samples from depths of 23 meters and 31 meters, respectively, at both the Three Poles and Willow Point sites. Briefly, the degenerative primers (IDT DNA) used were 5’GASGASACNACNACNGGNGT 3’ (SahH forward), 5’ TCVDWRTCRAARTGDCCRATRTT 3’ (SahH reverse), 5’ CATTATTAGATAGCTTTACADTNGAYCAYA 3’ (LuxS forward) and 5’ AGCGAGTGCATCTGATAAGWNCCRCAYTS 3’ (LuxS reverse). The letters N,S,R,V,D,W and Y all correspond to the standard UIB codes for degenerative bases [35]. All touchdown PCR reactions were performed as follows: initial denaturing at 95 °C for 5 min followed by the stepwise cycle of 95 °C for 30 seconds, 65 °C for 30 seconds and 72 °C for 1 minute. A total of 40 cycles were completed and the annealing temperature was decreased by 0.5 °C per cycle to reach a final annealing temperature of 45 °C. Final elongation was carried out at 72 °C for 5 minutes, and the reaction was held at +4 °C until separation on a 1.5% TBE (tris-borate-ethylene diamene tetra-acetic acid) agarose gel. Touchdown PCR was used to help circumvent some of the problems of normal PCR, such as mispriming of regions outside the target template. These misprimed alternate products can then outcompete the target product of the PCR reaction. There are several ways to overcome the problem of misprimed products, such as increasing the annealing temperature of the reaction and adjusting the magnesium concentration. However, both of these solutions are time-consuming and costly. Touchdown PCR is both a cost and time-effective way to maximize the intended reaction product. In touchdown PCR, the process is started with an annealing temperature well above the calculated Tm (melting temperature) of the used primers. Then, with every step or cycle of the PCR reaction, the annealing temperature is reduced, until the calculated Tm is reached (or negatively exceeded). Any difference between the correct Tm and the incorrect Tm will provide a two-fold per cycle (four-fold per °C) advantage to the correct product [36]. Touchdown PCR has also been shown to increase the specificity, sensitivity and efficiency of PCR reactions (even those reactions where the correct Tm is known) [37,38]. 

**Table 2:** Microbial signaling systems (adapted from Visick and Fuqua [29]).

Signal(s)	Microbe(s)	Synthesis	Precursor(s)	Receptor(s)	Regulated function(s)
Acyl homoserine lactones (AHLs)	*Proteo-bacteria*	-LuxI-type enzymes	-SAM, acyl-ACP	-LuxR-type proteins	Various processes
		-AinS-type enzymes	-SAM, acyl-ACP, acyl-CoA	-two-component kinase	
Linear oligopeptides	Gram-positive organisms	Genetically encoded	Secreted prepeptide	Two-component systems, phosphorelays	Various processes
Cyclical oligopeptides	Gram-positive organisms	Genetically encoded	Secreted post-syn mod. Prepeptide	Two-component systems, phosphorelays	Virulence genes
γ-Butyro-lactones (GBLs)	*Streptomyces* spp.	AfsA-type GBL synthase	Acyl-ACP, glycerol	ArpA-type repressor, GBL-binding proteins	Secondary metabolism; antibiotics and sporulation
Furanosyl diester (+/- boron); AI-2	Diverse taxa (no a- *proteo- bacteria)*	LuxS AI-2 synthase	Methionine slavage (DPD)	*Vibrio* spp. LuxP/LuxQ/LuxO phosphorelay; unknown for other systems	Luminescence and diverse processes
*Cis*-11-Methyl-2-dodecenoic acid (DSF)	*Xanthomonas* spp., perhaps others	RpfB,RpfF	Ayl-CoA	RpfC, RpfH	Virulence and pigmentation
4-Hydroxy-2-alkyl quinolines (PQS,HAQs)	Pseudomonads	pqs *ABCDE,* pqs*H*	Anthranilic acid	Unknown	Global regulation, virulence

## 3. Discussion and Conclusions

The distinctive assemblage of freshwater calcite microbialites at Pavilion Lake has been associated with organisms, such as *Epiphyton* and *Girvanella*, fossils from just before the Cambrian explosion about 550 million years ago [2]. The morphological diversity observed in the microbialite structures is astounding and ranges from mound-like structures to chimneys and large artichoke-type structures. Even more puzzling is that the structures have seemingly functional properties, most dominantly being displayed by the single internal conduit within the chimney structures (Figure 2), which is reminiscent of the structure of a sponge, the most ancestral of multicellular organisms. Yet, the structures are built by microbes only, and over 90% of the associated biomass is bacteria, based on Q-PCR analyses. Eukarya and archaea are at a comparatively low abundance in young, growing sections of the microbialites. The diversity of microbes populating the microbialites is very high and even includes sulphate reducing and anaerobic metal reducing bacteria (Figure 5a). The microbial diversity in the microbialites is much greater than the diversity observed in the lake and ground water samples. The biomass is also much higher in the microbialites, by at least one order of magnitude compared to the surrounding lake water. Yet, the microbialites are growing slowly; the microbial community members are in a stationary growth phase and have a low turnover rate, but do not indicate any evidence of starvation based on PLFA results. 

Analysis of two microbialite locations (Three Poles and Willow Point) at opposite sides of the basin shows a distinct difference in the microbial community. Ground-water seepage and nutrient inflow from a stream at Willow Point potentially affect the microbial population at these two sites. However, quorum sensing molecules were identified at both locations, indicating that quorum sensing may constitute a universal way for communication between the different microbial species involved in the build-up of the microbialites. We are only at the beginning of this promising research, and it will be fascinating to elucidate what microbial organisms are intrinsically involved in the build-up of these aggregates and how they are able to communicate and build these structures with apparently functional properties. What is already clear, however, is that the freshwater calcite microbialites at Pavilion Lake consist of a complex community of microorganisms that collectively form large, ordered structures, suggestive of aggregate functions similar to those of more complex colonial community ecosystems. Thus, they may represent a prototypical stage in the evolution of complexity wherever life has emerged. 

## References

[1] Lim D.S.S., Laval B.E., Salter G., Antoniades D., Forrest A.L., Pike W., Pieters R.,  Saffari M., Reid D., Schulze-Makuch D., Andersen D., McKay C.P. (2009). Limnology of Pavilion Lake, B.C., Canada—Characterization of a microbialite forming environment. Fundam. Appl. Limnol..

[2] Laval B., Cady S.L., Pollack J.C., McKay C.P., Bird J.S., Grotzinger J.P., Ford D.C., Bohm H.R. (2000). Modern freshwater microbialite analogues for ancient dendritic reef structures. Nature.

[3] Brady A.L., Slater G.F., Omelon C.R., Southam G., Druschel G., Andersen D.T., Hawes I., Laval B., Lim D.S.S. (2010). Photosynthetic isotope biosignatures in laminated micro-stromatolitic and non-laminated nodules associated with modern, freshwater microbialites in Pavilion Lake, B.C. Chem. Geol..

[4] Lim D.S.S., Brady A.L., Abercromby A.F., Andersen D.T., Andersen M., Arnold R.R., Bird J.S., Bohm H.R., Booth L., Cady S.L., Cardman Z., Chan A.M., Chan O., Chénard C., Cowie B.R., Davila A., Deans M.C., Dearing W., Delaney M., Downs M., Fong T., Forrest A., Gernhardt M.L., Gutsche J.R., Hadfield C., Hamilton A., Hawes I., Hansen J., Heaton J., Imam Y., Laval B.L., Lees D., Leoni L., Looper C., Love S., Marinova M.M., McCombs D., McKay C.P., Mireau B., Mullins G., Nebel S.H., Nuytten P., Pendery R., Pike W., Pointing S.B., Pollack J., Raineault N., Reay M., Reid D., Sallstedt T., Schulze-Makuch D., Seibert M., Shepard R., Slater G.F., Stonehouse J., Sumner D.Y., Suttle C.A., Trembanis A., Turse C., Wilhelm M., Wilkinson N., Williams D., Winget D.M., Winter C. (2011). A historical overview of the Pavilion Lake Research Project—Analog science and exploration in an underwater environment. Geol. Soc. Am..

[5] Lim D.S.S., Harwood C., Sumner D., Omelon C., Nienow J., Russell J., Biddle J., Brady A.L., Reid D., McKay C.P. (2012). Deep water microbialites of Pavilion Lake, Canada. Geobiology.

[6] Brady A., Slater G.F., Laval B., Lim D.S.S. (2009). Constraining carbon sources and growth rates of freshwater microbialites in Pavilion Lake using ^14^C analysis. Geobiology.

[7] White D.C., Bobbie R.J., Nichols J.S., Davis W.M., Fazio S.D. (1980). Nonselective biochemical methods for the determination of fungal mass and community structure in estuarine detrital microflora. Botanica marina.

[8] Gregory A.D. (2005). Microbial Insights.

[9] Edlund A., Nichols P.D., Roffey R., White D.C. (1985). Extractable and lipopolysaccharide fatty acid and hydroxy acid profiles from Desulfovibrio species. J. Lipid Res..

[10] Dowling N.J.E., Widdel F., White D.C. (1986). Phospholipid ester-linked fatty acid biomarkers of acetate-oxidizing sulfate reducers and other sulfide-forming bacteria. J. Gen. Microbiol..

[11] Parker J.H., Smith G.A., Fredrickson H.L., Vestal J.R., White D.C. (1982). Sensitive assay, based on hydroxy-fatty acids from lipopolysaccharide lipid A for gram negative bacteria in sediments. Appl. Environ. Microbiol..

[12] Bhat R.U., Carlson R.W. (1992). A new method for the analysis of amide-linked hydroxy fatty acids in lipid-A from gram-negative bacteria. Glycobiology.

[13] Smith G.A., Nickels J.S., Kerger R. (1986). Quantitative characterization of microbial biomass and community structure in subsurface material: a prokaryotic consortium responsive to organic contamination. Can. J. Microbiol..

[14] Schulze-Makuch D., Kennedy J.F. (2000). Microbiological and chemical characterization of hydrothermal fluids at Tortugas Mountain Geothermal Area, southern New Mexico, USA. Hydrol. J..

[15] Schulze-Makuch D., Goodell P., Kretzschmar T., Kennedy J.F. (2003). Microbial and chemical characterization of a groundwater flow system in an intermontane basin of southern New Mexico, USA. Hydrol. J..

[16] Kenyon C.N. (1972). Fatty acid composition of unicellular strains of blue-green algae. J. Bacteriol..

[17] Kenyon C.N., Rippka R., Stanier R.Y. (1972). Fatty acid composition and physiological properties of some filamentous blue-green algae. Arch. Microbiol..

[18] Caudales R., Wells J.M., butterfield J.E. (2000). Cellular fatty acid composition of cyanobacteria assigned to subsection II, order *Pleurocapsales*. Int. J. Syst. Evol. Microbiol..

[19] Fang J., Chan O., Joeckel R.M., Huang Y., Wang Y., Bazylinski D.A., Moorman T.B., Clement B.J.A. (2006). Biomarker analysis of microbial diversity in sediments of a saline groundwater seep of Salt Basin, Nebraska. Org. Geochem..

[20] Bühring S.I., Smittenberg R.H., Sachse D., Lipp J.S., Golubic S., Sachs J.P., Hinrichs K.-U., Summons R.E. (2009). A hypersaline microbial mat from the Pacific Atoll Kiritimati: Insights into composition and carbon fixation using biomarker analyses and a ^13^C-labeling approach. Geobiology.

[21] Guckert J.B., Antworth C.P., Nichols P.D., White D.C. (1985). Phospholipid, ester-linked fatty acid profiles as reproducible assays for changes in prokaryotic community structure of estuarine sediment. FEMS. Microbiol. Ecol..

[22] Guckert J.B., Hood M.A., White D.C. (1986). Phospholipid, ester-linked fatty acid profile changes during nutrient deprivation of *Vibrio cholerae*: increases in the *trans/cis* ratio and proportions of cyclopropyl fatty acids. Appl. Environ. Microbiol..

[23] Heipieper H.J., Diefenbach R., Keweloh H. (1992). Conversion of cis unsaturated fatty acids to trans, a possible mechanism for the protection of phenol degrading *Pseudomonas putida* P8 from substrate toxicity. Appl. Environ. Microbiol..

[24] Tsitko I.V., Zaitsev G.M., Lobanok A.G., Salkinoja-Salonen M.S. (1999). Effect of Aromatic Compounds on Cellular Fatty Acid Composition of *Rhodococcus opacus*. Appl. Environ. Microbiol..

[25] White D.C., Ringelberg D.B., Amy P.S., Haldeman D.L. (1997). Utility of the signature lipid biomarker analysis in determining the in-situ viable biomass, community structure and nutritional/physiologic status of deep subsurface microbiota. The Microbiology of the Terrestrial Deep Subsurface.

[26] Balkwill D.L., Leach F.R., Wilson J.T., McNabb J.F., White D.C. (1988). Equivalence of microbial biomass measures based on membrane lipid and cell wall components, adenosine triphosphate and direct counts in subsurface aquifer sediments. Microb. Ecol..

[27] Foster J.S., Green S.J., Ahrendt S.R., Golubic S., Hetherington K.L., Bebout L. (2009). Molecular and morphological characterization of cyanobacterial diversity in the stromatolites of Highborne Cay, Bahamas. ISME J..

[28] Goh F., Allen M.A., Leuko S., Kawaguchi T., Decho A.W., Burns B.P., Neilan P.A. (2009). Determining the specific microbial populations and their spatial distribution within the stromatolite ecosystem of Shark Bay. ISME J..

[29] Visick K.L., Fuqua C. (2005). Decoding microbial chatter: Cell-cell communication in bacteria. J. Bacteriol..

[30] Fuqua W.C., Winans S.C., Greenberg E.P. (1994). Quorum sensing in bacteria: the LuxR-LuxI family of cell density-responsive transcriptional regulators. J. Bacteriol..

[31] Whitehead N.A., Barnard A.M., Slater H., Simpson N.J., Salmond G.P. (2001). Quorum-sensing in Gram-negative bacteria. FEMS Microbiol. Rev..

[32] Smith J.N., Ahmer B.M. (2003). Detection of other microbial species by Salmonella: expression of the SdiA regulon. J. Bacteriol..

[33] Wagner-Döbler I., Thiel V., Allgaier M., Bodor A., Meyer S., Ebner S., Hennig A., Pukall R., Schulz S. (2005). Discovery of complex mixtures of novel long-chain quorum sensing signals in free-living and host-associated marine alphaproteobacteria. Chembiochem.

[34] Sun J., Daniel R., Wagner-Döbler I., Zeng A.P. (2004). Is autoinducer-2 a universal signal for interspecies communication: A comparative genomic and phylogenetic analysis of the synthesis and signal transduction pathways. BMC Evol. Biol..

[35] NC-UIB; Nomenclature for Incompletely Specified Bases in Nucleic Acid Sequences. http://www.chem.qmul.ac.uk/iubmb/misc/naseq.html.

[36] Don R.H., Cox P.T., Wainwright B.J., Baker K., Mattick J.S. (1991). Touchdown PCR to circumvent spurious priming during gene amplification. Nucleic Acids Res..

[37] Hecker K.H., Roux K.H. (1996). High and Low annealing temperatures increase both specificity and yield in touchdown and stepdown pcr. Biotechniques.

[38] Korbie D.J., Mattick J.S. (2008). Touchdown PCR for increased specificity and sensitivity in PCR amplification. Nat. Protoc..

